# Clinical Utility of SPECT Neuroimaging in the Diagnosis and Treatment of Traumatic Brain Injury: A Systematic Review

**DOI:** 10.1371/journal.pone.0091088

**Published:** 2014-03-19

**Authors:** Cyrus A. Raji, Robert Tarzwell, Dan Pavel, Howard Schneider, Michael Uszler, John Thornton, Muriel van Lierop, Phil Cohen, Daniel G. Amen, Theodore Henderson

**Affiliations:** 1 UCLA Medical Center, Los Angeles, California, United States of America; 2 The Synaptic Space, Denver, Colorado, United States of America; 3 University of British Columbia School of Medicine, Vancouver, British Columbia, Canada; 4 PathFinder Brain SPECT, Deerfield, Illinois, United States of America; 5 Sheppard Associates, Toronto, Ontario, Canada; 6 St. Johns Health Center, Santa Monica, California, United States of America; 7 Rossiter-Thornton Associates, Toronto, Ontario, Canada; 8 Private Practice, Toronto, Ontario, Canada; 9 Lions Gate Hospital, Vancouver, British Columbia, Canada; 10 Amen Clinics, Inc., Newport Beach, California, United States of America; Institute of Automation, Chinese Academy of Sciences, China

## Abstract

**Purpose:**

This systematic review evaluated the clinical utility of single photon emission computed tomography (SPECT) in traumatic brain injury (TBI).

**Methods:**

After defining a PICO Statement (Population, Intervention, Comparison and Outcome Statement), PRISMA (Preferred Reporting Items for Systematic Reviews and Meta-Analyses) criteria were applied to identify 1600 articles. After screening, 374 articles were eligible for review. Inclusion for review was focus on SPECT in the setting of mild, moderate, or severe TBI with cerebral lobar specificity of SPECT findings. Other inclusion criteria were comparison modalities in the same subjects and articles in English. Foreign language articles, SPECT studies that did not include comparison modalities, and case reports were not included for review.

**Results:**

We identified 19 longitudinal and 52 cross-sectional studies meeting inclusion criteria. Three longitudinal studies examined diagnostic predictive value. The first showed positive predictive value increases from initial SPECT scan shortly after trauma to one year follow up scans, from 59% to 95%. Subsequent work replicated these results in a larger cohort. Longitudinal and cross sectional studies demonstrated SPECT lesion localization not detected by CT or MRI. The most commonly abnormal regions revealed by SPECT in cross-sectional studies were frontal (94%) and temporal (77%) lobes. SPECT was found to outperform both CT and MRI in both acute and chronic imaging of TBI, particularly mild TBI. It was also found to have a near 100% negative predictive value.

**Conclusions:**

This review demonstrates Level IIA evidence (at least one non-randomized controlled trial) for the value of SPECT in TBI. Given its advantages over CT and MRI in the detection of mild TBI in numerous studies of adequate quality, and given its excellent negative predictive value, it may be an important second test in settings where CT or MRI are negative after a closed head injury with post-injury neurological or psychiatric symptoms.

## Introduction

TBI is a complex clinical phenomenon lacking a rigorously specified taxonomy, clear natural history, or pathoanatomical diagnostic criteria. The classic designations of mild, moderate, or severe TBI are based on the acute presentation and do not necessarily predict the long-term outcome. Moreover, the long-held assumption that the mild forms of this condition recover rapidly and without consequence is not supported by the more recent literature [1], [2]. The effects of several mechanisms for TBI (including impact, rotational and angular acceleration, and shear forces) lead to neurophysiological changes, cellular depolarization, and apoptosis that occur on a continuum and can progress over a protracted period of time [1]. The injuries associated with blast exposure often involved multiple mechanisms and may result in diffuse progressive brain damage [3]. It is now understood that those with mild TBI, particularly repetitive mild TBI, can have underlying neuropathology, that contributes to long-term increases in morbidity and mortality [1], [2], [4]–[6]. As the extent of undiagnosed or undertreated mild TBI becomes more evident [7], the endeavor of identifying TBI, particularly mild TBI, and thus providing effective treatments becomes increasingly important.

TBI affects both civilian and military populations. In 2003, the U.S. Centers for Disease Control and Prevention estimated the incidence of civilian TBI at 1.5 million [8]. Globally, this number is estimated at closer to 10 million [9]. Specific groups afflicted by TBI include an estimated 135,000 individuals per year from sports related concussion alone and 82 per 100,000 of employees of the transportation industry [10]. Meanwhile, the U. S. Department of Defense reported that over 266,000 soldiers experienced TBI between the years 2000–2012 [11]. The cost of TBI in the United States alone is considerable, estimated at over 76 billion dollars per year in 2000 [12]. Data released from the Congressional Budget Office showed that in the U.S. military, costs of TBI-related care are $11,700 per patient in the first year of treatment compared to $2,400 per year in patients with no TBI [13].

In addition to the financial costs of TBI, the long-term decline in health of persons with TBI is considerable. The rates of depression, anxiety, suicidality, drug and alcohol abuse, personality disorders, and other psychiatric symptoms are markedly elevated in survivors of TBI [2], [14]–[23]. For example, elderly persons with a history of TBI have a higher risk for cognitive decline and potentially for Alzheimer’s disease than peers without a history of the affliction [24], [25]. Repetitive mild TBI, also known as “repetitive concussion” [26], can lead to a progressive tauopathy known as chronic traumatic encephalopathy (CTE) [27]. There also is evidence of increased risk of homelessness [28] and higher rates of criminal behavior [29], [30].

The diagnosis of TBI, particularly mild TBI, remains a challenge clinically. There is a lack of gold standard neuropathological criteria to compare new diagnostic methods, although CTE shows promise [31]. Clinical presentation can also be confounded by the considerable overlap between the symptoms of mild TBI and posttraumatic stress disorder (PTSD). These overlapping symptoms can include headache, dizziness, irritability, sleep disturbances, sensitivity to light and noise, impulsivity, judgment problems, visual disturbances, emotional outbursts, depression, and anxiety. As in PTSD, neuropsychological impairments are common in TBI including memory impairment, delayed problem solving, slowed reaction time, fatigue, and impulsivity [32]–[34]. Such complexity can subsequently lead to misdirected treatment efforts, and can hamper the ability to accurately assess treatment response.

Neuroimaging remains a key focus of efforts to identify reliable changes in brain function that can lend insight into diagnosis and treatment of neurological diseases. Such techniques can be broadly divided into structural and functional techniques. Changes in brain structure represent a late change in most neurological disorders, such as dementia, when pathological cascades are often too advanced to optimize treatment [35]. As a consequence, structural changes may be insensitive to earliest changes seen in disease progression [36]. In TBI, this principle was illustrated in a recent study showing how changes in cerebral blood flow, a metric of brain function preceded changes in diffusion tensor imaging indicators of brain structure [37]. Additionally, cerebral perfusion abnormalities can persist even in chronic stages of TBI [38]–[41]. Functional imaging methods such as Single Photon Emission Computed Tomography (SPECT) can identify early changes in neurological diseases such as dementia by imaging regional cerebral blood flow, thus providing a predictive indicator of damage [42]. SPECT is of particular interest for such use because: i) it is a well-studied modality that has been previously utilized in such neurological disorders as epilepsy [43] and dementia [36]; ii) it has continuously seen hardware improvements from one head to three head cameras and from analog to digital detector components and; iii) it gains additional post-processing power with 3-D renderings and statistical analysis. Whether SPECT can yield such utility in the complex clinical setting of TBI is a question of great interest.

**Figure 1:**
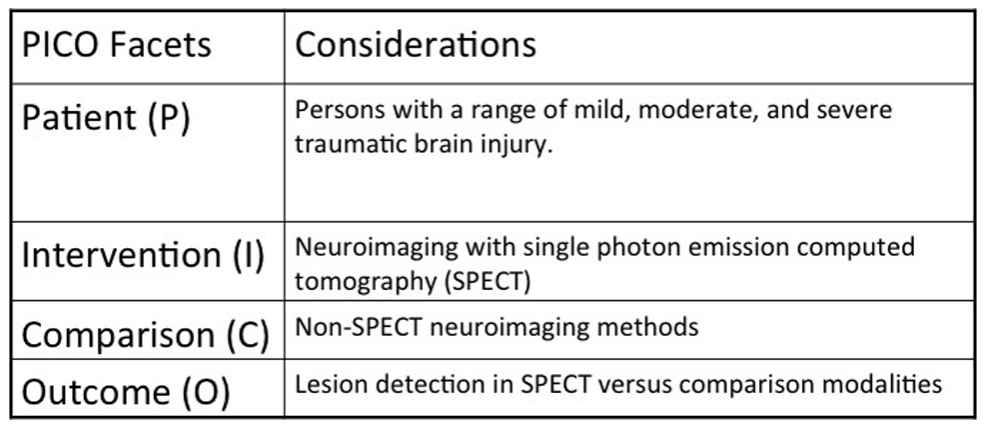
This figure describes the different components of a PICO statement and they are Patient, Intervention, Comparison, and Outcome. The second column describes each of these aspects for the current study.

The purpose of this systematic review is to evaluate the clinical relevance of SPECT in TBI by reviewing literature over the past 30 years. Figure 1 shows the Patient/Intervention/Comparison/Outcome (PICO) statement. We searched for randomized controlled trials (RCTs) and longitudinal cohort studies evaluating whether SPECT can identify TBI, focusing on the general anatomical lobar distributions of such deficits. We then identified from these same studies comparisons between identification of abnormalities in TBI on SPECT relative to other commonly utilized modalities such as CT and MRI to fulfill the goals of our PICO statement. In a secondary analysis, longitudinal cohort studies were also assessed for associations between SPECT abnormalities and neuropsychological and neurological outcomes. As a tertiary objective, we further characterized these relationships in eligible cross-sectional studies.

## Methods

### Search Strategy

We conducted a systematic review in accordance with the 2009 Preferred Reporting Items for Systematic Reviews and Meta-Analyses (PRISMA) guidelines [44]. With the consultation of an experienced health sciences librarian, a search of PubMed and Ovid MEDLINE was done in November of 2012. This was done using a series of search terms based upon the following Medical Subject Headings (MeSH) terms:

(“Tomography, Emission-Computed, Single-Photon” [MeSH] OR spect[tiab] OR “single photon emission computed tomography” [tiab] OR “Technetium Tc 99m Exametazime” [MeSH] OR hmpao[tiab] OR ecd[tiab] OR “Technetium Tc 99m Bicisate” [Supplementary Concept] OR “Cerebrovascular Circulation” [MeSH] OR “regional cerebral blood flow” [tiab] OR rcbf[tiab]) AND (“brain injuries” [MeSH] OR tbi[tiab] OR “traumatic brain injury” [tiab] OR concussion[tiab]) NOT (animals[MeSH] NOT humans[MeSH]).

Citations were imported into EndNote 6 (Thomson Reuters, New York, NY). The combined database yielded 1573 articles while an additional 27 articles were extracted from manual reference search selection. There was no duplication.

### Study Selection

Three authors reviewed all articles for inclusion with disagreements being resolved by discussion between reviewers. Longitudinal and cross-sectional studies were considered more important than case reports as the former study designs can track changes in patient populations over time and test relationships between variables of interest whereas case reports are susceptible to a higher magnitude of bias. Inclusion criteria for final review were: i) primary research articles published after 1983 to reflect more recent advances in SPECT imaging; ii) studies specific to SPECT application in persons with TBI; iii) Longitudinal cohort studies, RCTs, and cross-sectional studies; iv) full-text articles for evaluation of all study components and; v) studies in English or with available English translation. Exclusion criteria were: i) case series or case reports; ii) studies lacking a description of the lobar distribution of SPECT abnormalities; iii) and studies in a foreign language for which English translation was not available or feasible.

### Data Extraction and Quality Assessment

The article reviewers independently extracted the following data: number of participants, study recruitment setting, type of SPECT tracer used, and medical/neurological/psychiatric co-morbidities if available. In longitudinal studies, cohort age mean or range and gender were also acquired. For all studies, lesion localization on SPECT at a lobar level (frontal, temporal, parietal, occipital, and cerebellum) was noted. Studies that had neuropsychological or neurological outcomes were identified and any statistically significant correlations between perfusion abnormalities on SPECT imaging and these tests were noted.

We also identified the duration between sentinel TBI events and time of SPECT scan for cross-sectional studies. Additional variables categorized were TBI definitions on a category of mild, moderate, and severe as defined by each study. Quality of longitudinal studies was assessed using the Newcastle-Ottawa Scale [45] of which 8 was the highest possible score in this review. Data extraction and categorization was done using Statistical Package for Social Science (SPSS, version 20.0, IBM, Armonk, NY).

## Results

**Figure 2:**
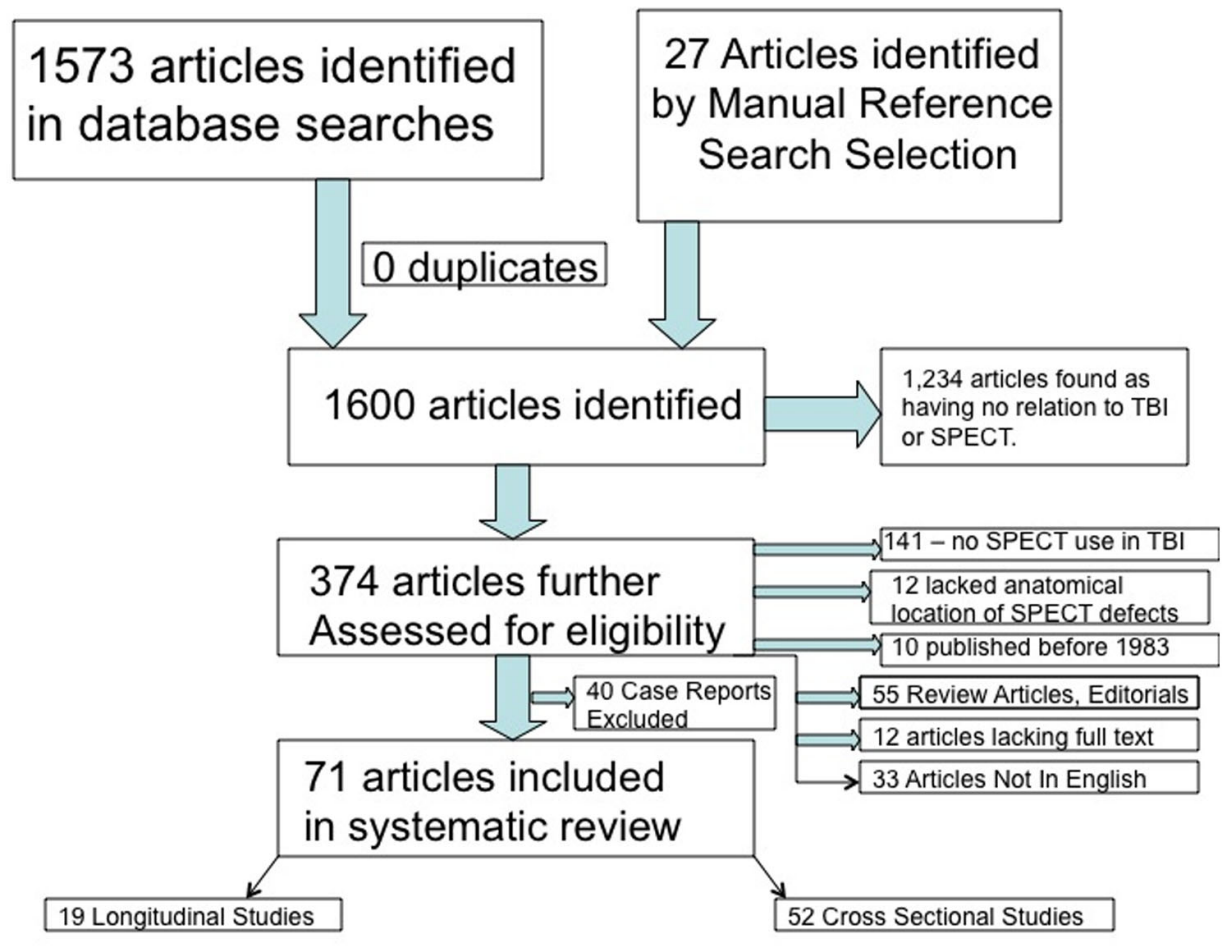
This figure outlines a flowchart of article selection in this study from the initial 1600 that were identified to the final 71 manuscripts that were included in the systematic review.

The initial database literature search yielded 1,600 potential articles (Figure 2), including 27 identified by manual reference search. After the original phase of screening, 374 articles were obtained for full-text review. During full-text screening, 296 additional studies were disqualified. Of the 71 articles remaining, seven were found to have considerable overlapping of cohorts but were included for analysis after they were assessed to have evaluated separate questions compared to other articles using the same cohort. When considering these, this study overviewed 2,634 unique persons with TBI through 30 years of compiled literature.

### Summary of Longitudinal Studies

A total of 19 longitudinal analyses met all inclusion criteria for the main analysis and included five intervention studies (Table 1; [41]; [46]–[53]). A total of 903 persons were assessed in these studies with NOS scores between 4 to 8. With respect to general trends, 13 studies (68%) had 657 persons including otherwise healthy subjects while a smaller proportion of studies (15%) included subjects with medical, neurological, or psychiatric co-morbidities. There were 13 studies (68%) in which SPECT scans were acquired months to years after the sentinel TBI event. Severe TBI was the most common type assessed, in 7 (37%) of studies followed by mild and moderate TBI (21%), mild TBI alone (10.5%), and all severities of TBI (16%). There were three studies (16%) where severity was not specifically defined. In terms of functional neuroimaging, 11 (58%) of the studies used 99mTc-HMPAO (hexamethylpropylene amine oxime) as the most common SPECT radiotracer, followed by Tc-99m ECD (ethyl cysteinate dimer) in 5 (26%) of studies with the remainder tracers either being xenon in two studies (10%) or not specifically described in 6% of studies. The common type of SPECT device used were one headed cameras in 6 studies (32%) followed by three headed cameras in 5 studies (26%) and two headed cameras in two studies (11%). The remaining six studies did not specifically describe the number of heads on the SPECT camera. Visual evaluations of SPECT scans were the most common type of analysis approach used in 9 (47%) of studies followed by quantitative assessment of SPECT scans with statistical parametric mapping, in 5 (26%) of papers and the remainder of methods used a combination of methods. With respect to lesion localization, the frontal lobes were the most commonly abnormal regions in 18 studies (95%) along with temporal in 18 (95%) studies, followed by parietal in 17 (89%) of studies, occipital in 16 (84%) studies and cerebellum in 14 (74%) studies. Ten of the longitudinal studies (52%) include comparison modalities to SPECT; both structural CT and MRI in 6 studies (32%) and structural CT alone in 4 (21%) of studies. SPECT identified abnormalities not seen on MRI and CT in all 10 (100%) of these studies. Of the 19 longitudinal studies, 14 of them (77%) had neurological or neuropsychology outcomes of which SPECT abnormalities correlated with such outcomes in 13 of them (93%). Specifically, SPECT perfusion changes were statistically significant in their association with neuropsychological or neurological tests. This included 2 out of 5 intervention trials (40%) correlating SPECT perfusion changes with improved neuropsychological or neurological outcomes.

**Table 1:** Summary of Longitudinal and Intervention Trials. Underline = Intervention.

Study/Year	N, Start/Follow Up	Age	Male Gender (%)	Lesion Localization SPECT,CT/MR	Follow Up, mos	NOS Score
Agarwal 2005 [46]	30/30	2–18	70	T	3	7
Amen 2011 [47]	100/100	57	100	F/T/P/O/C	6	7
Amorim 2011 [48]	7/7	37	86	F	NA	4
Barclay 1985 [49]	12/4	19	83	F/T/P	1.6	5
Barrett 2004 [50]	5/5	35	60	F/T/P/O/C	12	6
Bavetta 1994 [51]	10/10	29	80	F/T/P/O/C (Y)	17	7
Golden 2002 [52]	50/50	25	62	F/T/P/O/C	1	5
Gowda 2006 [53]	120/92	28	82	F/T/P/O/C (Y)	14	8
Harch 2012 [54]	16/16	30	100	F/T/P/O	1	8
Jacobs 1994 [55]	67/25	35	61	F/T/P/O (Y)	3	6
Jacobs 1996 [41]	136/73	36	62	F/T/P/O (Y)	6	7
Kaloostian 2012 [56]	120/120	35	NA	F/T/P/O (Y)	6	8
Laatsch 1997 [57]	3/3	30	33	F/T/P/O (Y)	45	4
Laatsch 1999 [58]	5/5	37	40	F/T/P/O (Y)	6	4
Lewis 2006 [59]	5/5	37	40	F/T/P/O/C (Y)	12	4
Mazzini 2003 [60]	140/140	36	81	F/T/P/O (Y)	12	6
Mitchener 1997 [61]	32/32	31	84	F/T/P/O (Y)	6	7
Newton 1992 [62]	19/19	29	79	F/T/P/O (Y)	3–36	6
Shiina 1998 [63]	26/26	29	NA	F/T/P/O (Y)	1.4	5

This table describes a summary of longitudinal and intervention trials included in this systematic review. Underlined first authored names denote intervention studies. The column on Lesion Localization denotes lobar distributions of SPECT abnormalities described in the evaluated articles with F = Frontal lobe, T = temporal lobe, P = Parietal lobe, O = Occipital lobe, and C = cerebellum. The (Y) denotes a yes to answer the question if a given evaluated paper described abnormalities on SPECT not visualized or described on comparison modality imaging. The column marked NOS denotes the Newcastle-Ottawa Scale score assigned for each longitudinal study or intervention article. Paper citations are integrated into the table.

### Longitudinal Diagnostic Predictive Value

Three longitudinal studies examined specific metrics of diagnostic predictive value. Jacobs et. al. [55] used SPECT to prospectively evaluate patients with mild (N = 25) or moderate (N = 42) TBI. Each patient had a clinical evaluation and a SPECT scan within four weeks of the initial injury and three months after the first scan. Of the 33 patients who showed no significant abnormalities on their initial SPECT scan, 97% of the patients resolved their clinical symptoms within three months. By contrast, of the 34 patients who had abnormalities on their first SPECT scan, 59% of the patients continued to experience significant clinical symptoms. The positive predictive value of an abnormal initial scan was only 20/34 (59%), but if the second scan three months later was also abnormal the sensitivity for the repeat SPECT was 19/20 (95%). These authors suggest that negative initial SPECT studies can be a reliable predictor of a favorable clinical outcome. In a subsequent study, Jacobs [41] evaluated the predictive capacity of HMPAO SPECT for clinical outcome during a follow-up period of 12 months after mild head injury. They prospectively evaluated 136 patients with mild head injury who underwent initial SPECT imaging within 4 weeks after the trauma (93% within two weeks of injury). All patients with an abnormal initial SPECT underwent a repeat SPECT study at 2.9–3.3 months, 5.7–6.3 months, and 11.9–12.6 months post-injury. Patients with a previously normal SPECT scan did not undergo a repeat study. Clinical reassessments were performed over the subsequent 12 months as long as the prior SPECT scan was positive or until patients were completely asymptomatic. During all follow-up evaluations, SPECT had a high sensitivity and negative predictive value, increasing from 91% and 89%, respectively, at 3 months to 100% at 6 months and at 12 months. At 12 months post-injury, the authors observed considerable improvement in the specificity and positive predictive value of SPECT (85% and 83%, respectively). In a recent longitudinal study by Kaloostian et al. [56] of 120 patients suffering from severe TBI, as defined by a Glasgow Coma Scale (GCS) <8, Receiver Operating Curve (ROC) Data for SPECT predicting GCS scale at 6 months for cerebral perfusion measured at <6 and <12 hours after sentinel TBI was 92% and 77%.

### Summary of Cross Sectional Studies

A total of 52 studies met inclusion and exclusion criteria for analysis (Table 2 [38]; [64]–[114]). This includes a combined sample size of 2,121 persons with TBI. Regarding general observations, severe TBI was the most common type of TBI studied, in 17 (33%) studies, followed by mild TBI in 10 (19%) of studies. There were 12 (23%) studies that examined all severities of TBI, mild, moderate and severe. There were 17 (33%) studies in which persons with TBI were imaged months to years after the sentinel event. Still, 12 (23%) of the studies entailed imaging patients days after TBI. As with longitudinal studies, the frontal lobe was the most commonly abnormal region identified, in 49 (94%) of studies. This was followed by the temporal lobe in 40 (77%) of studies, parietal lobe in 38 (74%) of studies, occipital lobe in 27 (52%) of studies, and the cerebellum in 13 (25%) of studies. In 36 of the studies, structural CT and MRI were the most common comparison modalities. Of the studies assessing such comparisons, 98% of such studies showed SPECT lesion localization not identified by structural imaging or that was larger in size than suggested by structural lesions. Of the 22 studies that assessed neuropsychological relationships between SPECT lesion localization and neuropsychological tests, 18 (81%) of them demonstrated a statistically significant correlation with SPECT visualized lesion.

**Table 2:** Summary of Cross Sectional Studies.

Study Year	TBI Type	SampleSize	ComparisonImaging	Time of SPECT	LesionLocalization	Lesion Detection Not Seenon Comparison Imaging
Abdel-Dayem 1998 [64]	Mild and Moderate	228	SPECT Only	Months After TBI	F/T/P	Not applicable
Abe 2003 [65]	Severe TBI	80	CT and MRI	Days to weeks	F	Yes
Abu-Judeh 1999 [66]	Mild TBI	32	CT Structure	Months After TBI	F/T/P/O	Yes
Abu-Judeh 2000 [67]	Mild,Moderate	228	CT Structure	Months After TBI	F/T/P/O	Yes
Amen 2011 [68]	Mild, Moderate	100	SPECT Only	Years after TBI	F/T/P/O/C	Not applicable
Assadi 2007 [69]	All severities	92	CT and MRI	Not Mentioned	F/T/P/O/C	Yes
Audenaert 2003 [70]	Mild TBI	8	CT Structure	Days after TBI	F/P/O	Yes
Beuthien- Baumann2003 [71]	Severe TBI	16	FDG PET	Months to Years	F/P/O	Yes
Bicik 1998 [72]	Other Definition	13	MR and FDG PET	Days after TBI	F	Yes
Bonne 2003 [38]	Mild TBI	28	CT and MRI	Years after TBI	F/T/O	Yes
Choksey 1991 [73]	Severe TBI	8	CT Structure	Not Mentioned	F/T/P	Yes
Cusumano 1992 [74]	Severe TBI	68	CT Structure	Days after TBI	F/P	Yes
Donnemiller 2000 [75]	All severities	10	CT and MRI	Months to Years	F	Yes
Ducours 1990 [76]	Mild and Severe	20	CT Structure	Days after TBI	F/P	Yes
Eftekhari 2005 [77]	Mild, Moderate	14	SPECT Only	Years after TBI	F/O	Not applicable
Emanuelson 1997 [78]	All severities	20	CT Structure	Years after TBI	F/T/P/O/C	Yes
Goethals 2004 [79]	Severe TBI	57	CT and MRI	Months After TBI	F/P	Yes
Goldenberg 1992 [80]	Severe TBI	36	SPECT Only	Months to Years	F/T	Not applicable
Goshen 1996 [81]	Severe TBI	28	CT and MRI	Not Mentioned	F/T/P/O/C	Yes
Gray 1992 [82]	All severities	53	CT Structure	Months After TBI	F/T/P/O/C	Yes
Hashimoto 2009 [83]	Mild TBI	9	SPECT Only	Not Mentioned	F	Not applicable
Hattori 2009 [84]	Mild TBI	30	SPECT Only	Years after TBI	F/T/P/C	Not applicable
Hofman 2001 [85]	Mild TBI	21	MR Structure	Days after TBI	F/T/P	Yes
Ichise 1994 [86]	All severities	29	MR Structure	Not Mentioned	F/T/P/O	Yes
Ito 1997 [87]	Severe TBI	8	MR Structure	Days to months	F/T/P/O/C	Yes
Jian 2009 [88]	Severe TBI	16	CT and MRI	Not Mentioned	None	Yes
Kant 1997 [89]	Mild TBI	43	CT and MRI	Months to Years	F/T/P	Yes
Kauppinen 2002 [90]	Mild TBI	18	SPECT Only	Days after TBI	F	Not applicable
Kemp 1995 [91]	Mild and Moderate	32	SPECT Only	Not Mentioned	F/T/P/O	Not applicable
Kesler 2000 [92]	All severities	52	MR Structure	Months to Years	F/T	No
Kinuya 2003 [93]	All severities	35	CT and MRI	Days after TBI	F/T/P/O/C	Yes
Korn 2005 [94]	Mild TBI	17	CT and MRI	Months to Years	F/T/P/O	Yes
Laurin 1989 [95]	All severities	18	SPECT Only	Days after TBI	F/T/P/O	Not applicable
Lewine 2007 [96]	Mild TBI	58	MEG and MRI	Months to Years	F/T/P/O	Yes
Lorberboym 2002 [97]	Mild, Moderate	16	CT Structure	Hours after TBI	F/T/P/O	Yes
Loutfi 1995 [98]	Not Defined	12	SPECT Only	Not Mentioned	F/T/P/O	Not applicable
Mann 2006 [99]	Severe TBI	6	CT and MRI	Months to Years	F/T/P	Yes
Mazzini 2003 [100]	Severe TBI	143	MR Structure	Months After TBI	F/T	Yes
Nagamachi 1995 [101]	All severities	23	CT Structure	Days to months	F/T/P/O/C	Yes
Oder 1992 [102]	Severe TBI	36	SPECT Only	Months After TBI	F/T	Not applicable
Okamoto 2007 [103]	Severe TBI	27	MR Structure	Months to Years	F/T/C	Yes
Reid 1990 [104]	Severe TBI	13	CT Structure	Days after TBI	F/T/P/O	Yes
Roper 1991 [105]	All severities	15	CT Structure	Days after TBI	F/T/P/O	Yes
Rupright 1996 [106]	Severe TBI	6	CT and MRI	Not Mentioned	P/T/O	Yes
Sakas 1995 [107]	All severities	53	CT and MRI	Weeks after TBI event	F/T	Yes
Sataloff 1996 [108]	Not Defined	191	CT and MRI	Days after TBI	F/T/P/O/C	Yes
Shin 2006 [109]	All severities	13	MR Structure	Weeks after TBI event	F/T/P	Yes
Silverman 1993 [110]	Not Defined	2	MR Structure	Not Mentioned	O	Yes
Umile 1998 [111]	Moderate TBI	4	SPECT Only	Months to Years	F/T/P/O	Not applicable
Wiedmann 1989 [112]	Moderate TBI	16	CT and MRI	Months to Years	F/T/P	Yes
Wong 2006 [113]	Severe TBI	8	SPECT Only	Years after TBI	F/T/P/O/C	Not applicable
Yamakami 1993 [114]	Severe TBI	12	CT Structure	Days after TBI	F/T/P	Yes

This table describes a summary of cross sectional studies included in this systematic review and include columns on sample size, lobar distribution, relative lesion identification on SPECT compared to other modalities, when SPECT imaging took place, and classification of TBI. Paper citations are integrated into the table.

### Intervention Trials

Longitudinal studies have also demonstrated that cerebral blood flow on SPECT can be used as a biomarker and surrogate endpoint for evaluating effectiveness of new treatments. Laatsch et al. [57], [58] studied 5 patients who had acquired brain injury and initially demonstrated neuropsychological deficits and various degrees of hypoperfusion on SPECT. Following cognitive rehabilitation therapy (CRT) all clients were able to return to productive employment or schooling. Examination of the neuropsychological testing results revealed significant improvement in performance following CRT that was generally maintained after treatment. SPECT data revealed that, in a majority of cases, significant increases in relative cerebral blood flow redistribution was also seen.

In a recent study by Harch et al. [54], 16 military subjects who had received mild to moderate TBI via blasts, underwent neuropsychological evaluation, and then received 40 HBOT sessions over 30 days. The HBOT was at 1.5 atmospheres of oxygen. Neuropsychological evaluations completed within one week after treatment demonstrated an increase of 14.8 IQ points (p<0.001) as well as improvements in depression and anxiety indices. Additionally, quantitative analysis of SPECT scans showed improvement in blood flow. While the findings of this article were considered controversial by some [115], we included it in our review as the study authors extensively addressed such concerns in separate published correspondence [116]. Amen and colleagues [47] showed how a multifactorial lifestyle and dietary supplement intervention program was related to improved blood flow on SPECT and performance on tests of neuropsychological function in a cohort of retired American Professional Football players. Areas showing improved perfusion with intervention were the prefrontal cortex, anterior cingulate gyrus, precuneus, occipital lobes, and cerebellum.

## Discussion

This systematic review identified a considerable body of literature establishing a relationship between SPECT and: i) improved lesion detection in TBI compared to typical comparison modalities such as CT and MRI; ii) neuropsychological and neurological outcomes; iii) and treatment interventions. These findings suggest that SPECT should be part of a clinical evaluation in the diagnosis and management of TBI, a concept articulated in work by other groups [117]. We identified 19 longitudinal studies that demonstrate Level II A evidence, evidence from at least one controlled trial without randomization, supporting the utility of SPECT as a key modality for identifying lesions in the clinical setting of TBI [118]. That the majority of these studies were able to demonstrate these findings on lower resolution one-headed cameras suggests that newer SPECT devices and post-processing methods may hold greater sensitivity to detecting TBI, as has been described for the detection of early dementia [119], [120]. A key implication of such work is that SPECT can identify deficits associated with sub-acute and chronic TBI. The longitudinal studies include intervention trials that also suggest the utility of cerebral blood flow on SPECT as a potential biomarker for surrogate endpoints in assessing the effectiveness of new treatments.

The 52 cross sectional studies we identified also support the clinical utility of SPECT suggested by longitudinal studies. For example, Lewine et al. [96] identified that the odds ratio for the predictive value of a SPECT abnormality was 2.3 for psychiatric complaints, 5.7 for somatic complaints, and 1.5 for cognitive complaints, superior to structural MR imaging. Only MEG was better than SPECT in one category - cognitive complaints. However, many of these studies are susceptible to confounding as they lacked baseline SPECT scans for comparison. By nature of their design, cross sectional studies are also vulnerable to confounding by unmeasured variables. Additionally as definitions and classifications of TBI have evolved over time, comparing different varieties of TBI across studies is non-standardized and therefore another unavoidable limitation in the current literature.

Both longitudinal and cross sectional studies provided insight into lesion localization in TBI. In both types of studies, the frontal lobes were the most commonly affected region. This finding has implications for anatomical localization in clinical practice, vulnerability to other psychiatric disorders such as PTSD that are also associated with frontal lobe dysfunction, and determining risk for neurocognitive deficits in such domains as executive function [121]. The findings of temporal lobe hypoperfusion in longitudinal studies as being equal to the frontal lobes in terms of frequency of abnormalities lends insight as to why persons with TBI have increased risk for Alzheimer’s disease [122].

SPECT can assist in the diagnosis, prognosis, and treatment of patients who have sustained brain trauma. It is conceivable that SPECT may also uncover occult brain trauma in clinically confusing or complex cases as reported symptoms can range in specificity and frequency [123]. SPECT may also reveal occult TBI in cases of treatment resistant or treatment-unresponsive conditions, for example depression [124], [125]. Indeed, the American College of Radiology suggests certain situations in which SPECT may be useful in TBI assessment as a problem solving modality for complex cases or in acute and sub-acute groups, particularly if CT or MRI are non-contributory [126] as directly quoted below:

“SPECT studies may reveal focal areas of hypoperfusion that are discordant with findings of MRI or CT [53]–[56]. On the basis of these results, some investigators suggest that these functional imaging techniques may explain or predict postinjury neuropsychologic and cognitive deficits that are not explained by anatomic abnormalities detected by MRI or CT [51]–[52], [54]. Furthermore, focal lesions demonstrated by SPECT offer objective evidence of organic injury in patients whose neuroimaging studies are otherwise normal [52].”

While it is logical to utilize rapidly attainable structural scans such as non-contrast CT scans for acute TBI in the emergency room setting, the questions remains as to how to best diagnose and treat patients for which TBI is often a chronic, if clinically subtle, entity in sub-acute and chronic populations. If, as the reviewed data suggest, perfusion SPECT has a negative predictive value near 100%, a negative scan is diagnostically and prognostically important after a head injury with psychiatric sequelae. Differentiating between mild TBI and psychological reaction to head injury is difficult clinically, particularly when CT and MRI are normal. Furthermore, new onset difficulties with affect regulation, impulse control and interpersonal function may be outside the ability of psychological tests to link to TBI, because tests typically focus on cognitive domains and lack etiological specificity. The persistence or even progression of symptoms despite normal morphological imaging and psychological testing is clinically common. Alternatively, an abnormal perfusion SPECT, according to these data, has higher sensitivity than CT or MRI. TBI is now thought to possibly reflect a progressive, inflammatory neurological injury, even when overlooked or dismissed in subclinical cases. An individual with subclinical TBI which only becomes clinically manifest months or years after injury may be misdiagnosed and therefore suboptimally treated, along with being denied legitimate benefits or services. This scenario could be greatly simplified with a positive baseline scan which shows or does not show progression, in concert with clinical findings and test results. The positive initial scan may also prompt more aggressive clinical intervention to prevent progression of the pathophysiologic process, even in the absence of clinical symptoms, with the potential to completely alter the patient’s life trajectory. An overall approach is to use clinical assessment of TBI patient signs and symptoms to select who should receive SPECT scans to more sensitively screen for brain functional defects. This strategy could be applied in persons with recent or history of remote trauma to guide treatment and rehabilitation. Future studies should attempt to ascertain the clinical utility and effectiveness of such models. Future studies could address the role of other functional modalities, such as functional MRI, Positron Emission Tomography (PET), or combination modalities such as PET-CT or the more recent PET-MRI in acute and chronic clinical settings [127], [128].

A possible limitation of this review is that we did not overview case reports or gray literature such as conference abstracts. However, this decision was made to allow for assessment of only the most high quality literature in order to most accurately characterize data and trends in the field of SPECT neuroimaging in TBI. Consequently, this work represents a rigorous overview of SPECT as applied to TBI. It is important to note that many imaging modalities for most conditions whether they are chest radiographs for pneumonia, mammograms for breast cancer, or SPECT for TBI, can primarily provide sensitivity in the detection of a pathological state and that further clinical assessment and tests are paramount to offering specificity in a diagnosis. A mastectomy is not planned based on mammogram results alone; rather, needle biopsy and clinical examination guide treatment. Similarly, the use of SPECT imaging in TBI would have to be utilized as a way of providing sensitivity to the diagnosis while other tools of clinical assessment would add to specificity. Developing such clinical tools should also remain a goal of future research. It is important to note that, while CT and MRI are relatively insensitive for TBI in comparison with SPECT, unlike SPECT, they offer considerably greater specificity, due to high-resolution depiction of in vivo morphology. Not all perfusion defects are TBI, and we would be remiss if we did not point out that diagnostic imaging of the brain is incomplete without morphological examination.

Another potential limitation is that the studies did not all report, nor did they all conform to, one single standard for performing brain perfusion imaging. Relevant differences that might go unreported could include whether study subjects were in a resting state or performing a concentration task, and if in a resting state, whether the injection room was dark and quiet, plus how long subjects were left in the resting state prior to injection. Even so, the discrete perfusion deficits of TBI may not be affected by the concentration state of the brain, nor the presence of external stimuli during the injection phase. Thus, we cannot say with certainty whether this is an important limitation, though we suspect it may not be [129].

A final limitation worth considering is whether different tracers with different biokinetics might influence the accuracy of SPECT. Because 99mTc-ECD and 99mTc-HMPAO have high extraction fractions and rapid blood clearance, with little back-diffusion and a 6 hour physical half-life for 99mTc, they are considered “static” tracers. 133Xe, being chemically inert, remains lipophilic on either side of the neuronal membrane, and back diffusion is relevant, so it is considered a “dynamic” tracer in the context of rCBF imaging. It also has non-ideal imaging properties, principally a low imaging energy of 80 keV and rapid exchange in tissues, which result in poorer count statistics and thus decreased spatial resolution. So, in theory, it may be less sensitive for TBI. Nonetheless, our review did not uncover any direct comparisons of 133Xe with either 99mTc-ECD or 99mTc-HMPAO, so the difference remains unproven [130].

In conclusion, the current state of literature demonstrates both associative and predictive value of SPECT in the setting of TBI. This same literature also demonstrates certain advantages of SPECT compared to structural MRI and CT in multiple studies, particularly in mild TBI. SPECT can therefore be used to provide actionable information in the identification and management of TBI.

## Supporting Information

Checklist S1 This checklist identifies portions of this manuscript that are linked to specific items in the PRISMA checklist system.
(DOC)
